# The Fate of Osteoblast-Like MG-63 Cells on Pre-Infected Bactericidal Nanostructured Titanium Surfaces

**DOI:** 10.3390/ma12101575

**Published:** 2019-05-14

**Authors:** Jason V. Wandiyanto, Vi Khanh Truong, Mohammad Al Kobaisi, Saulius Juodkazis, Helmut Thissen, Olha Bazaka, Kateryna Bazaka, Russell J. Crawford, Elena P. Ivanova

**Affiliations:** 1 School of Science, Faculty of Science, Engineering and Technology, Swinburne University of Technology, Hawthorn, VIC 3122, Australia; jwandiyanto@swin.eud.au (J.V.W.); makobaisi@swin.edu.au (M.A.K.); 2 School of Science, College of Science, Engineering and Health, RMIT University, Melbourne, VIC 3000, Australia; vi.khanh.truong@rmit.edu.au (V.K.T.); olga.bazaka@rmit.edu.au (O.B.); russell.crawford@rmit.edu.au (R.J.C.); 3 Center for Micro-Photonics, Faculty of Science, Engineering and Technology, Swinburne University of Technology, Hawthorn, VIC 3122, Australia; Sjoudkazis@swin.edu.au; 4 CSIRO Manufacturing, Clayton, VIC 3168, Australia; helmut.thiessen@csiro.com; 5 Institute for Future Environments, Queensland University of Technology, GPO Box 2434, Brisbane, QLD 4001, Australia; katerina.bazaka@qut.edu.au

**Keywords:** mechano-bactericidal surfaces, competitive colonization, titanium, MG-63

## Abstract

Biomaterials that have been newly implanted inside the body are the substratum targets for a “race for the surface”, in which bacterial cells compete against eukaryotic cells for the opportunity to colonize the surface. A victory by the former often results in biomaterial-associated infections, which can be a serious threat to patient health and can undermine the function and performance of the implant. Moreover, bacteria can often have a ‘head start’ if implant contamination has taken place either prior to or during the surgery. Current prevention and treatment strategies often rely on systemic antibiotic therapies, which are becoming increasingly ineffective due to a growing prevalence of antibiotic-resistant bacteria. Nanostructured surfaces that kill bacteria by physically rupturing bacterial cells upon contact have recently emerged as a promising solution for the mitigation of bacterial colonization of implants. Furthermore, these nanoscale features have been shown to enhance the adhesion and proliferation of eukaryotic cells, which is a key to, for example, the successful osseointegration of load-bearing titanium implants. The bactericidal activity and biocompatibility of such nanostructured surfaces are often, however, examined separately, and it is not clear to what extent bacterial cell-surface interactions would affect the subsequent outcomes of host-cell attachment and osseointegration processes. In this study, we investigated the ability of bactericidal nanostructured titanium surfaces to support the attachment and growth of osteoblast-like MG-63 human osteosarcoma cells, despite them having been pre-infected with pathogenic bacteria. MG-63 is a commonly used osteoblastic model to study bone cell viability, adhesion, and proliferation on the surfaces of load-bearing biomaterials, such as titanium. The nanostructured titanium surfaces used here were observed to kill the pathogenic bacteria, whilst simultaneously enhancing the growth of MG-63 cells in vitro when compared to that occurring on sterile, flat titanium surfaces. These results provide further evidence in support of nanostructured bactericidal surfaces being used as a strategy to help eukaryotic cells win the “race for the surface” against bacterial cells on implant materials.

## 1. Introduction

Bacterial contamination of implanted biomaterials is difficult to prevent or mitigate [1,2], and there are only few strategies available that can achieve this effectively without compromising the compatibility of the material with host cells. Examples of successful strategies include sol-gel synthesized hydroxyapatite gel coatings doped with silver [3] and zinc [4,5], enamel-like coatings fabricated using thermal evaporation [6], surface functionalization using nonthermal atmospheric pressure nitrogen plasmas [7], Sharklet™-like grooves and grid micro-structures imparted on the surfaces via ultra-short pulsed laser ablation [8], and others. In the context of the contest between human cells trying to integrate with the implant material and bacteria attempting to establish a biofilm on a substratum, microorganisms often have the advantage that they can have contact with the implant material, contaminating it prior to or during surgery. As a result, the bacteria are in a better position to win “the race for the surface” [9]. This often leads to biomaterial-related infections, which are difficult or impossible to eradicate using systemic antibiotics, particularly in cases where the biofilm is already established and/or when the surface is infected with antibiotic-resistant bacteria [10,11,12]. If left untreated, such biofilms present a serious health concern, with potential consequences ranging from acute and chronic inflammatory responses and associated bone loss and soft tissue damage, to sepsis and increased risk of morbidity and mortality [13,14]. Biofilms on implants can also act as a source of chronic infection. For load bearing implants, inflammation and presence of bacteria and their endotoxins in the osseous milieu may interfere with the homeostasis between osseous tissue formation and resorption [15,16], maintained by osteoblasts and osteoclasts, respectively [17]. Indeed, inflammatory cytokines, e.g., interleukin-1 (IL-1) or tumor necrosis factor alpha (TNF-α), and lipopolysaccharides of Gram-negative bacteria may promote the proliferation of osteoclastic precursors, as well as differentiation or maturation of macrophages and multinucleated bone-resorbing cells that have been shown to be responsible for low-grade bone resorption [18,19,20]. Inflammation-related local changes in pH, temperature and ion concentration may affect or reverse adsorption of proteins, such as fibronectin, vitronectin, and other extracellular matrix (ECM) proteins that play an important role in chemotactic, mechanical, and adhesive stimulation of biomineralization and matrix maturation [21].

In the context of bacteria developing increasing resistance to antimicrobials drugs, bactericidal strategies based on physical, rather than chemical, mechanisms are becoming increasingly important [22,23]. Nanostructured bactericidal surfaces that employ a specific pattern of macro-, micro-, and nanoscale features to mechanically rupture and kill bacteria represent one of the best developed methods to achieve this goal. First discovered when observing the cell killing abilities of cicada and dragonfly wings [12,24,25] and later recreated artificially, a recent study on the interactions between a mechano-bactericidal nanostructured surface and antibiotic-resistant bacteria demonstrated that such surfaces were able to mechanically rupture the cell walls of both antibiotic-resistant and antibiotic-susceptible *Staphylococcus aureus* cells with similar levels of bactericidal efficiency. In parallel, nanostructured surfaces with increased degrees of surface roughness have been shown to improve adhesion and proliferation of eukaryotic cells, which is a key to successful osseointegration [26,27,28]. Bactericidal activity and biocompatibility of nanostructured surfaces are, however, often studied separately, which may not necessarily represent the entire spectrum of real-world surgical scenarios [29,30,31]. For example, it is not evident as to which extent bacterial cell-surface interactions would affect host cell attachment and osseointegration outcomes for implants that rely on mechano-bactericidal nanostructured surfaces to mitigate bacterial colonization. Hence, investigations on how nanostructured bactericidal surfaces could help eukaryotic cells compete and win against bacteria in “the race for the surface” on biomaterial implant materials is essential to further develop this technology.

Several in vitro and in vivo models have been developed to study the effects and the interaction between pathogenic bacteria and eukaryotic cells during the processes of simultaneous competitive colonization of implant surfaces. For example, in studies by Pérez-Tanoira et al., host cell viability and integration with an implant surface was found to strongly depend on the concentrations of bacteria present, with bacterial colonization being shown to be reduced in the presence of human cell attachment [32]. Similarly, Martinez-Perez et al. found that eukaryotic cells affected pathogenesis of implant-related infections and in turn, were affected by the presence of several staphylococci strains [33]. From these examples, it is evident that for bactericidal surfaces, such as those that employ mechanical rupture of bacteria, to be effectively translated into clinical applications, it is imperative to study the process of competitive colonization using suitable in vitro models. For this reason, in this work, we investigated the capability of bactericidal nanostructured titanium to promote successful integration of osteoblast-like MG-63 cells on the titanium surface pre-infected with two of the most common pathogenic bacteria encountered in hospital infections, *Staphylococcus aureus* and *Pseudomonas aeruginosa.*

## 2. Materials and Methods

### 2.1. Materials

Commercially pure (CP) American Society for Testing and Materials (ASTM) grade 2 titanium billets were used to manufacture Ti discs. Potassium hydroxide was purchased from Merck Millipore (Burlington, MA, USA). Analytical grade ethanol (Labtek Pty Ltd., Brendale, QLD, Australia) and acetone (Merck Millipore, Burlington, MA, USA) were used to clean the Ti discs. Methicillin and gentamicin were purchased from Sigma-Aldrich (St. Louis, MO, USA).

### 2.2. Preparation and Fabrication of Ti Samples

CP grade 2 Ti billets (*d* = 10 mm) were cut into discs with thickness of 2 mm, using a dicing machine (Secotom 50, Struers, GmbH, Willich, Germany). The discs were then polished, using silicon carbide grinding paper with a grit size of P1200. Prior to hydrothermal etching, Ti discs were further cleaned, using ultrasonic agitation in MilliQ water, 100% ethanol, 100% acetone, and 50% ethanol, in that order, for 6–8 min per cycle. The cleaned Ti discs were then allowed to dry at 37 °C for 24 h. The polished and cleaned Ti disks (referred to as AR-Ti) were used as control, non-modified planar titanium surfaces.

Ti surfaces were hydrothermally etched (referred to as HTE-Ti) following the protocol reported elsewhere [34], except in this work, 1 M KOH was used to etch Ti substrata. Prior to the experimental work, the HTE-Ti and AR-Ti were sterilized with 70% ethanol and UV-treated in a 1300 Series Class II Type A2 biological safety cabinet (Thermo Fisher Scientific, Waltham, MA, USA) for 0.5 h, this being a standard sterilization protocol for implants [35].

### 2.3. Bacterial Strains, Preparation, and Pre-Infection Procedure

Bacterial strains used in this study were methicillin- and gentamicin-susceptible *Staphylococcus aureus* CIP 65.8^T^, obtained from the Culture Collection of the Institute Pasteur (CIP, Paris, France), and *Pseudomonas aeruginosa* ATCC 9721 obtained from the American Type Culture Collection (ATCC, Manassas, VA, USA). Bacterial stocks were prepared in a 20% glycerol nutrient broth (Oxoid, Basingstoke, Hampshire, UK) and stored at −80 °C. Fresh bacterial suspensions were prepared for each of the strains at the infective dose of 10^5^ cells per mL for *S. aureus* and 10^3^ cells per mL for *P. aeruginosa* in phosphate buffered saline (PBS) (10 mM, pH 7.4). AR-Ti and HTE-Ti disks were immersed into bacterial suspensions and incubated for 6 h at 37 °C in dark conditions. All experiments were conducted with at least three independent technical replicates. 

### 2.4. Bactericidal Efficacy of Titanium Surfaces

Dynamic bactericidal efficacy was assessed using a standard plate count method following the protocol described elsewhere [36]. Pathogenic bacterial cells were suspended in PBS (10 mM, pH 7.4) and cell density was adjusted to the respective infectious dose according to Federal Drug Administration (FDA) guidelines [37]. Bactericidal efficiency was estimated as the decrease in the number of remaining viable cells relative to the control after 3 and 6 h.

### 2.5. Confocal Laser Scanning Microscopy

Confocal laser scanning microscopy (CLSM) was used to visualize the proportion of viable and non-viable bacteria on titanium surfaces. A 1 mL aliquot of bacterial suspension of each bacterial strain was placed on the surface of cleaned and sterilized AR-Ti and HTE-Ti discs and allowed to incubate for 18 h at 25 °C. After incubation, the discs were gently washed with copious amounts of sterile MilliQ water to remove non-attached cells following a previously developed experimental protocol [38]. Five samples of each surface type including controls were used for each strain. At least three independent technical replicates were performed to confirm the results.

The bacteria were stained using a LIVE/DEAD BacLight Bacterial Viability Kit, L7012 (Thermo Fisher Scientific, Waltham, MA, USA), which contained a mixture of SYTO^®^ 9 and propidium iodide fluorescent dyes. The SYTO^®^ 9 dye permeates viable and non-viable cells, binding to nucleic acids and fluorescing green when excited by a 485 nm wavelength laser. Propidium iodide is only capable of entering cells that have undergone significant membrane damage (and are therefore considered to be nonviable), binding with higher affinity to the nucleic acids present in the bacterial cells than the SYTO^®^ 9 dye and fluorescing red when excited by a 535 nm wavelength laser. Images were recorded using a Fluoview FV3000 microscope (Olympus, Tokyo, Japan) at 60× magnification with water immersed lens. Images were taken across ten different fields of view to get a representation of the entire sample.

The mammalian cell lines were stained with a LIVE/DEAD Viability/Cytotoxicity Kit (Invitrogen, CA, USA) composed of calcein AM and ethidium bromide with the excitation and emission waves of 494/517 nm and 528/617 nm, respectively. Prior to the assay, the samples were first washed with PBS and stained with the dye mixture for 15 min. Then, the samples were washed again with PBS before the assay.

### 2.6. Scanning Electron Microscopy

High-resolution electron micrographs were obtained using a field emission SEM (SUPR 40 VP, Carl Zeiss, Oberkochen, Germany) at 3 kV under 15,000× magnification. To assess cellular morphology of bacterial and MG-63 cells, titanium discs were sputter-coated with gold, using a Dynavac CS300 (Perogon Technologies, Wheelers Hill, VIC, Australia) prior to imaging.

### 2.7. MG-63 Cell Line

The MG-63 cell line was obtained from Sigma-Aldrich (USA) and was cultured in Dulbecco’s Modified Eagle’s medium (DMEM, Sigma-Aldrich, MO, USA), supplemented with 10% fetal bovine serum (FBS) and 1% penicillin/streptomycin (Sigma-Aldrich). All laboratory experiments were approved by the Biosafety Project 2014/SBC01. Cells were grown to reach 80% confluency and then were trypsinized using 0.25% trypsin/EDTA (Thermofisher, Waltham, MA, USA). Cells were seeded on pre-infected titanium surfaces at a density of 10,000 cells/cm^2^ for every independent experiment. All the following assessments were performed after 1, 3, and 5 days of seeding when 100% confluency was reached, and three independent technical replicates were performed for each type of surface.

### 2.8. Immunohistochemistry

To perform immunohistochemistry staining, cells were gently washed with PBS, fixed in 4% p-formaldehyde for 15 min, permeabilized in 0.1% Triton X for 5 min, then blocked with 1% bovine serum albumin (BSA) for 60 min. Image-IT® FX Signal Enhancer (Invitrogen) was also used during fixation to enhance fluorescent staining. Fixed cells were treated with primary antibody overnight, followed by secondary antibody. Actin filaments were visualized by staining the cells with Alexa Fluor 488 conjugated Phalloidin (Invitrogen). Nucleus were labeled using TO-PRO3 (Invitrogen). Samples with stained cells were then placed in a glass-bottomed disc for imaging.

### 2.9. MTS Cell Proliferation Assay

To perform the 3-(4,5-dimethylthiazol-2-yl)-5-(3-carboxymethoxyphenyl)-2-(4-sulfophenyl)-2H-tetrazolium (MTS) assay, the medium was removed, and the cells were washed with PBS solution. Then a fresh medium containing 10% of MTS solution (Promega, WI, USA) was added to the samples and they were incubated at 37 °C for 1.5 h. After the incubation period, the medium was transferred to a 96-well plate (Sarstedt, Nümbrecht, Germany) and the absorbance was recorded at 490 nm using Polarstar Omega microplate reader (BMG Labtech, Ortenberg, Germany).

### 2.10. Lactate Dehydrogenase (LDH) Cytotoxicity Assay

The supernatant of the medium was transferred to 96-well plate (Sarstedt, Nümbrecht, Germany) and LDH reaction mix (Promega) was added to the supernatant. Then the mixture was incubated for 30 min at room temperature (*ca*. 25 °C) in the dark. LDH stop buffer solution was added at the end of the incubation and the absorbance was recorded at 490 nm.

### 2.11. Bicinchoninic Acid Assay (BCA)

The medium was removed from the samples and the samples were washed with PBS once. Then mammalian protein extraction agent was added to the samples to extract the proteins out from the cells and incubated at room temperature (*ca*. 25 °C) for 15 min. After the incubation, the supernatant was then transferred to the 96-well plate (Sarstedt, Nümbrecht, Germany) with the added BCA (Sigma-Aldrich) working solution, prepared according to manufacturer’s instructions, and incubated at 37 °C for 30 min. The absorbance was recorded at 562 nm.

### 2.12. Real-Time Quantitative Polymerase Chain Reaction (qRT-PCR)

qRT-PCR was used to quantify the levels of gene expression for collagen type 1 (Col 1), alkaline phosphatase osteocalcin (ALP), and osteopontin (OPN), with glyceraldehyde 3-phosphate dehydrogenase (GAPDH) as a housekeeping gene to normalize the expression level. The primer sequences of each gene were obtained from Marinucci et al [39]. To prepare this experiment, MG-63 cells were seeded at 10,000 cells/cm^2^ and incubated for 10 days. After incubation, total RNA was extracted from the cells using TRIsure reagent (Bioline, London, UK), according to the manufacturer’s protocol. After RNA extraction, the samples were treated with DNase to eliminate any genomic DNA, and the remaining RNA was then converted to DNA. Prior to quantifying the samples, the quality of DNA was measured using Nanodrop (ThermoFisher, Waltham, MA, USA). Each RT-PCR was performed with the following reagents: 10 μL of 2× SensiFAST SYBR & Fluorescein Mix (Bioline), 200 ng of DNA in the samples, 1 μL of each forward and reverse primer (10 μM) and an appropriate volume of nuclease-free water to bring the total reaction volume to 20 μL. The reactions were prepared in 96-well plates sealed with optical quality sealing tape (Bio-Rad, Hercules, CA, USA). The reactions were analyzed using MyiQ^TM^ single-color detection system (Bio-Rad, USA) with the following cycling conditions: polymerase activation at 95 °C for 1 min, followed by 40 cycles of denaturation (95 °C for 15 s), primer annealing (54 °C for 30 s) and extension (72 °C for 15 s). Data were collected during the annealing step. A melt curve was obtained by increasing the set-point temperature from 60 °C to 95 °C with a step of 0.5 °C every 10 s. GAPDH was used as a housekeeping control. Differential gene expression fold change was calculated using 2^−ΔΔC^_T_ (Livak) method [40], where ΔΔC_T_ was calculated from the equation below, taking samples of MG-63 cells grown on AR-Ti as a calibrator. With the equation below, ΔC_T_ values are provided from real-time PCR instrumentation where it is the difference between the target gene and the reference gene (in this work is GAPDH).

ΔC_T(test)_ = C_T(target,test)_ − C_T(ref,test)_, (1)

ΔC_T(calibrator)_ = C_T(target,calibrator)_ − C_T(ref, calibrator)_, (2)

ΔΔC_T_ =ΔC_T(test)_ −ΔC_T(calibrator)_, (3)

Expression ratio = 2^−ΔΔC^_T_. (4)

## 3. Results and Discussion

### 3.1. Bactericidal Activity of Hydrothermally Treated Ti (HTE-Ti)

HTE-Ti surfaces have previously been shown to possess high bactericidal activity against pathogenic bacteria and even antibiotic-resistant strains [13,30,31,34]. Additionally, they are biocompatible with eukaryotic cells and have been shown to enhance their differentiation [31,41,42]. It remains unclear, however, whether these surfaces could still support the adhesion and proliferation of eukaryotic cells after being pre-infected with live pathogenic bacteria. In this study, HTE-Ti surfaces were inoculated with viable *S. aureus* CIP 65.8^T^ or *P. aeruginosa* ATCC 9721 for 6 h at their respective infective doses [43,44]. An incubation period of 6 h was allowed, as this period is known as the “decisive period” for medical-implant-associated infections and is considered critical for the initiation of infection [45]. The subsequent colonization of MG-63 cells on the pre-infected surfaces was then monitored for 1, 3, and 5 days to study the MG-63 cells attachment and proliferation. 

**Figure 1:**
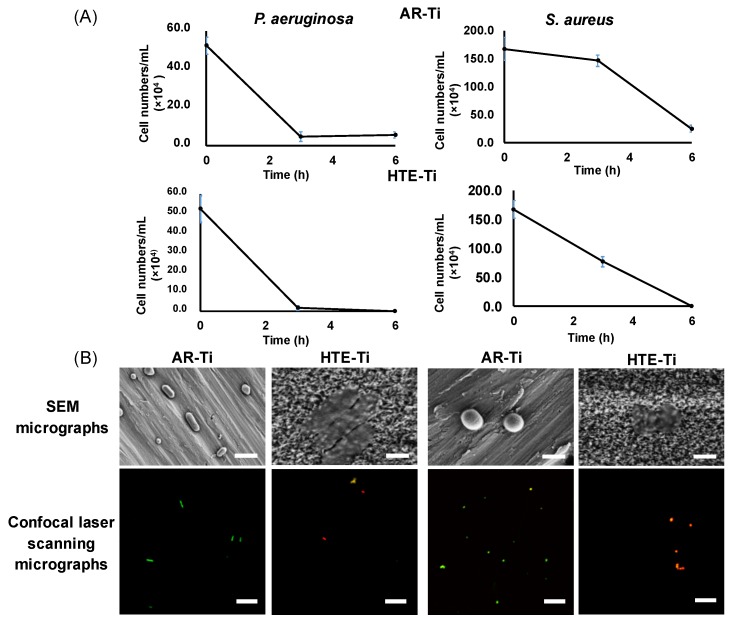
Attachment and viability of *P. aeruginosa* (left) and *S. aureus* (right) cells upon interaction with surfaces of smooth control (AR-Ti) and nanostructured hydrothermally treated titanium (HTE-Ti) surfaces. (**A**) Rapid reduction in cell viability confirms bactericidal activity of HTE-Ti against *P. aeruginosa* (left) and *S. aureus* (right) over 6 h incubation. (**B**) Representative SEM and Confocal laser scanning microscopy (CLSM) micrographs showing the altered morphology of bacterial cells attached onto HTE-Ti surfaces compared to the normal morphology of cells attached onto AR-Ti surfaces, and presence of live cells (stained green) on AR-Ti and dead cells (stained red) on HTE-Ti surfaces (CLSM Scale bar = 400 nm, SEM scale bar = 1 µm).

The results of examination of pre-infected titanium surfaces after 6 h indicated that both species of pathogenic bacteria were successfully inactivated as confirmed through direct plate count technique (Figure 1), while viable bacterial cells remained on the AR-Ti surface. Visualization of attached bacterial cells using SEM and CLSM clearly indicated that the HTE-Ti nanostructured surfaces were able to physically rupture bacterial cells leaving only cell debris. In contrast, both strains of bacteria were able to maintain their cellular integrity on the AR-Ti surfaces.

The mechanism by which a nanostructured surface, such as that of HTE-Ti, kills bacteria is based on the physical rupturing of the bacterial cell membrane, which experiences mechanical stress as the cells attach onto the nanostructured surfaces [24,25]. This process was first observed when studying the attachment behavior of bacteria on the surfaces of cicada wings, where the surface could kill *P. aeruginosa* cells within a few minutes of direct contact [24]. While the nature of the forces involved in rupturing the bacterial cells remains poorly understood, it is well documented that Gram-negative bacteria are affected by such interactions to a greater degree than Gram-positive bacteria [25,46]. This increased resilience is due to the presence of a thick peptidoglycan layer in the wall of the Gram-positive bacteria, which increased the rigidity of the cells, rendering them less susceptible to mechanical rupture due to the membrane having the ability to stretch and sag across the surface. This effect is also shown in Figure 1A, where the killing rate against *P. aeruginosa* cells is seen to be greater than the killing rate against *S. aureus* cells. Within 3 h of incubation, most *P. aeruginosa* cells were inactivated by HTE-Ti surfaces, however, it took significantly longer to kill the majority of the *S. aureus* cells. Although *S. aureus* cells have greater rigidity than the *P. aeruginosa* cells, both bacterial strains experienced mechanical stress due to direct contact with the nanostructures that led to the rupturing of the cell membranes at the end of the 6 h incubation period, as shown in Figure 1B.

### 3.2. Ability of Eukaryotic Cells to Proliferate on Pre-Infected HTE-Ti

**Figure 2:**
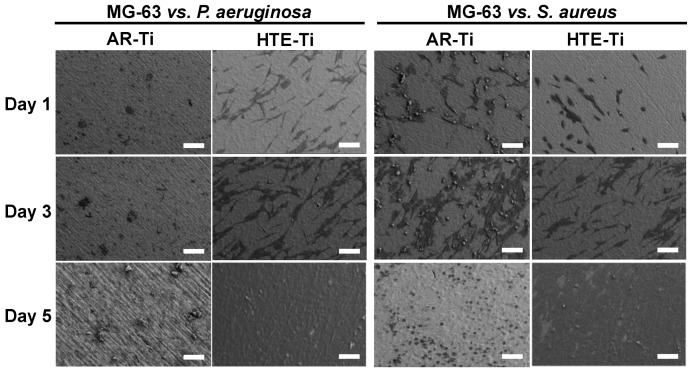
Attachment and proliferation of MG-63 cells on pre-infected titanium surfaces during a 5-day incubation period. Representative SEM images show that on the HTE-Ti surfaces, cells were able to grow successfully, achieving confluency over a 5-day incubation period, whereas the growth of the MG-63 cells on flat, titanium surfaces (AR-Ti) was inhibited due to the colonization of the surface by pathogenic bacteria (scale bar = 20 µm).

**Figure 3:**
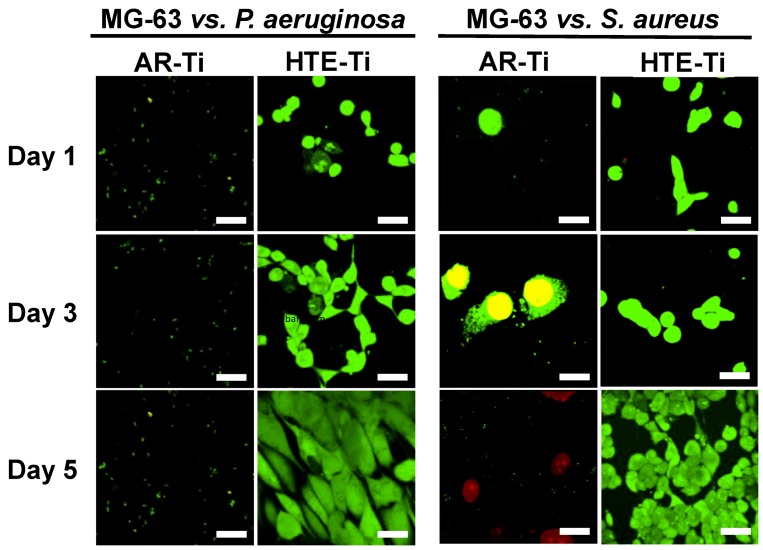
Typical CLSM images of MG-63 cells over a 5-day incubation period on pre-infected nanostructured HTE-Ti and smooth AR-Ti titanium surfaces. Cells on the HTE-Ti surfaces were observed to grow successfully over a 5-day incubation period, showing a healthy morphology and an elongation associated with tissue formation. Growth of the MG-63 cells on the flat, titanium surface (AR-Ti) was inhibited due to the presence of pathogenic bacteria, with cells adopting a round morphology and reduced viability. Scale bar = 5 µm.

The subsequent colonization of MG-63 cells on pre-infected titanium surfaces showed that these eukaryotic cells were able to attach and proliferate on the pre-infected HTE-Ti surfaces to 100% confluency over 5 days. The MG-63 cells were able to attach onto both the HTE-Ti and AR-Ti surfaces on day 1 (Figure 2). Cells on the HTE-Ti surface were also able to elongate, forming bipolar and sometimes multipolar shaped cells, while the cells on the AR-Ti surfaces were circular in their shape, even after 5 days of incubation. Through CLSM visualization (shown in Figure 3), it could be seen that the viability of the MG-63 cells was found to be strongly affected by the presence of *P. aeruginosa* cells on the AR-Ti surfaces from day 1. Comparatively, the MG-63 cells were viable and could coexist with the *S. aureus* cells on the AR-Ti surface until day 3. Although the MG-63 cells were viable on the AR-Ti surfaces until day 3, many of these cells still retained their spherical shape. In contrast, on the HTE-Ti surfaces, the MG-63 cells were able to elongate to form their bipolar morphology from day 1.

The morphology, attachment, and viability of the MG-63 cells on the pre-infected surfaces were studied after 1, 3, and 5 days of incubation (Figure 3). The seeded MG-63 cells appeared to be viable and could proliferate to 100% confluency by day 5 over the HTE-Ti surfaces, despite these surfaces being pre-infected with live pathogenic bacteria. In contrast, on flat AR-Ti surfaces, both bacterial strains remained viable after 6 h of incubation and were able to overwhelm and kill the MG-63 cells despite the presence of antibiotics (1% penicillin/streptomycin) in the culture medium used to cultivate MG-63 cells. The SEM and CLSM images in Figure 2 and Figure 3 showed that the MG-63 cells were unable to attach and grow on the AR-Ti surfaces infected with *P. aeruginosa* cells as the bacterial cells were able to grow, starving the osteoblast-like cells from the nutrients, killing the MG-63 cells on day 1, as a result. In contrast, the MG-63 cells were able to attach and grow over the AR-Ti surface infected with *S. aureus* cells until day 3. Eventually, the bacteria overgrew the surface, resulting in the eventual death of the MG-63 cells on day 5. *P. aeruginosa* cells are known to be more virulent than *S. aureus* cells, as they tend to secrete toxins when invading host cells [47,48]. Hence, the application of antibiotics in the medium was not enough to prevent the bacteria (at their infective doses) from growing over the surface, forming biofilms. In contrast, surfaces with bactericidal nanostructures were able to eliminate the bacterial contamination and ensure that the cell successfully attached to the surface, confirming their potential to be used as tissue integration promoters in biomaterials.

**Figure 4:**
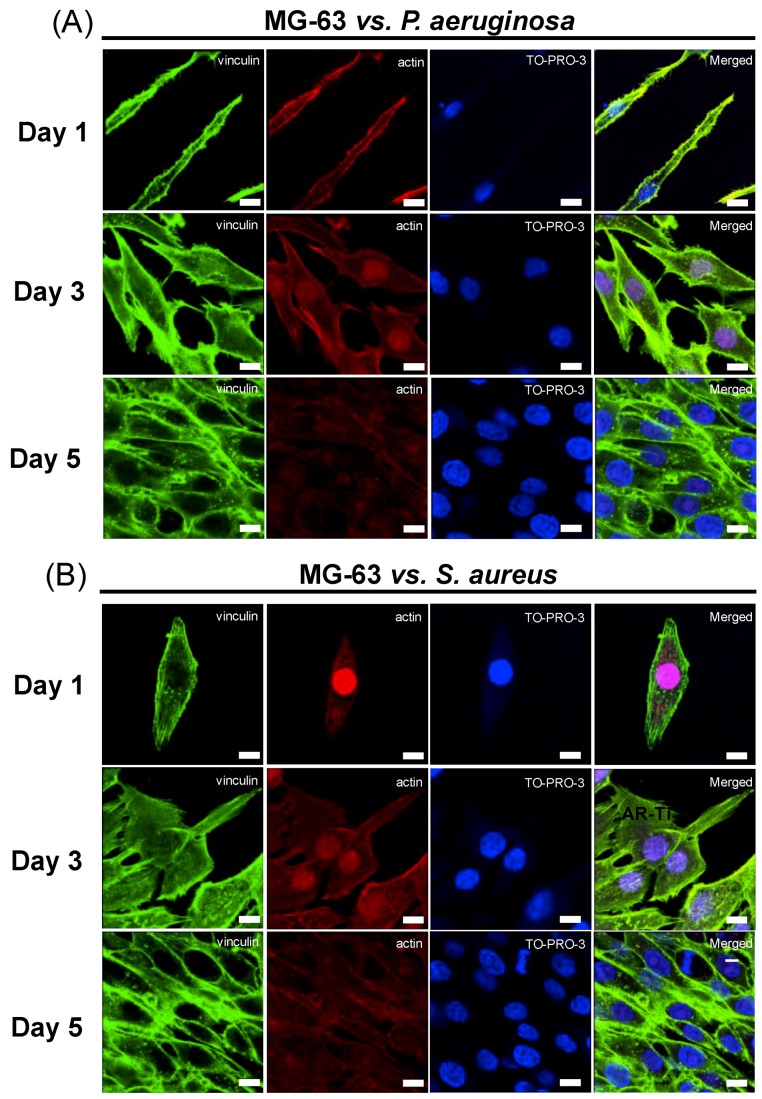
Immunohistochemical staining of proteins associated with growth and proliferation of MG-63 cells during their incubation on HTE-Ti surfaces pre-infected with (**A**) *Pseudomonas aeruginosa* and (**B**) *Staphylococcus aureus* cells. Vinculin is stained green, actin is red, and TO-PRO3 is blue. Cells were able to adhere to the pre-infected HTE-Ti surfaces and proliferate over the 5-day incubation period through the detection of the proteins responsible for attachment. (Scale bar = 20 µm).

Immunohistochemistry staining of vinculin, actin, and TO-PRO3 were performed on the osteoblast-like cells in order to visualize the expression of the proteins responsible for their attachment and growth, and to observe how these changed over the course of a 5-day incubation period. The MG-63 cells on the HTE-Ti surfaces pre-infected with either *P. aeruginosa* or *S. aureus* cells were able to express vinculin and actin, which are proteins known to be responsible for promoting cell attachment to surfaces. The images presented in Figure 4A,B show the results of immunohistochemistry staining of proteins associated with growth and proliferation of MG-63 cells during their incubation on HTE-Ti surfaces pre-infected with *P. aeruginosa* and *S. aureus* bacteria, respectively. Similar protein expression was demonstrated by MG-63 cells grown on sterile AR-Ti surfaces, which served as a control (Supplementary, Figure S1).

**Figure 5:**
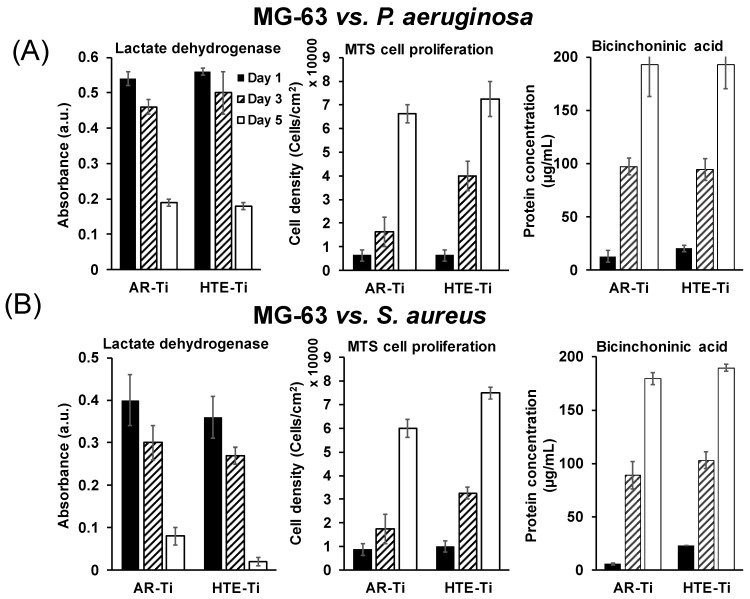
The growth of MG-63 cells on pre-infected surfaces of substrata with (**A**) *Pseudomonas aeruginosa* and (**B**) *Staphylococcus aureus.* The lactate dehydrogenase (LDH) assay, which indicates cell damage, showed that the cells had recovered after day 5, which was accompanied by an increase in the cell numbers (MTS cell proliferation assay) and total protein concentration (BCA), in response to their interaction with the pre-infected nanostructured surfaces.

Various bioassays, such as LDH, MTS cell proliferation, and BCA, were also used to further assess the wellbeing of the cells on the pre-infected surfaces. The data presented in Figure 5A,B show the results of these bioassays for the MG-63 cells grown on surfaces pre-infected with *P. aeruginosa* and *S. aureus* cells, respectively. The results of the LDH assay show that there is a decrease in LDH concentration from day 1 to day 5 of incubation of the MG-63 cells grown on both the pre-infected HTE-Ti and sterile AR-Ti surfaces, whereas a rapid increase of cell density and protein concentration on the MG-63 cells was observed on both surfaces. The cell density on the pre-infected HTE-Ti surface increased significantly compared to that observed for the cells on the AR-Ti surface on day 3. Additionally, the cells on the HTE-Ti surface possessed a slightly higher protein concentration on day 1 compared to that of the cells on the AR-Ti surface on day 1. Even though the HTE-Ti surfaces were infected with either *S. aureus* or *P. aeruginosa* cells, the bioassay results pertaining to the MG-63 cells exhibited no significant difference between these groups, as can be seen by the data presented in Figure 5A,B. This further demonstrated the ability of the HTE-Ti surface to neutralize the pathogenic bacteria upon contact with the surfaces, henceforth allowing the MG-63 cells to grow over the surface.

**Figure 6:**
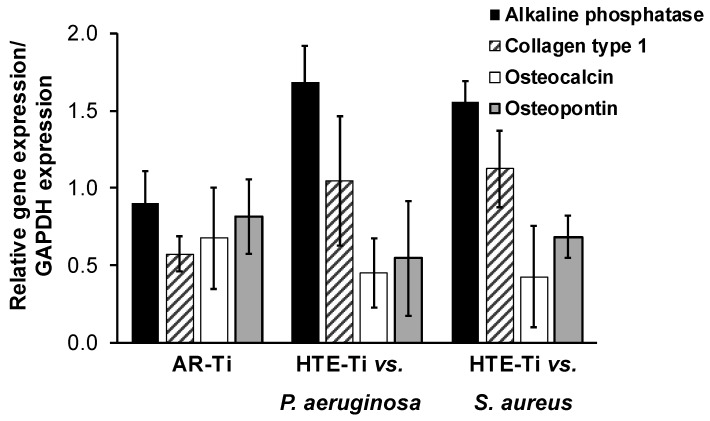
Expression of osteogenesis-related factors in MG-63 cells after 10 days of incubation on the nanostructured surfaces pre-infected with *Pseudomonas aeruginosa* and *Staphylococcus aureus* cells. The expressions of alkaline phosphatase—and collagen type 1 (Col 1)—were found to be greater on HTE-Ti surfaces, whereas osteocalcin and osteopontin (OPN) were expressed to a lesser extent on these surfaces. Glyceraldehyde 3-phosphate dehydrogenase (GAPDH) was used as a reference gene to normalize the expression levels.

The osteogenesis-related gene expression of the MG-63 cells was monitored using qRT-PCR to investigate the response of the MG-63 cells to the unique surface features present on the bactericidal HTE-Ti substratum. The gene expression quantification results presented in Figure 6 demonstrate that in general, the nanostructured surface upregulated the expression of target genes in the MG-63 cells, despite the substratum surfaces having been pre-infected with pathogenic bacteria. After a 10-day incubation period, the differentiation markers for alkaline phosphatase and COL 1 expressed by the MG-63 cells were found to be greater than those present on the sterile AR-Ti surface. These results provided further evidence as to the significantly greater ability of the nanostructured surfaces being able to simultaneously kill pathogenic bacteria, while simultaneously promoting MG-63 cell adhesion and growth, compared to that observed on the AR-Ti surfaces.

The results of the immunohistochemistry staining presented in Figure 4A,B suggests that proteins such as vinculin and actin could be detected by the cells, implying that they were able to attach to the HTE-Ti surfaces, despite the having been pre-infected with pathogenic bacteria. Vinculin and actin serve as focal adhesion proteins, and their presence is essential for successful cellular attachment. An intracellular focal adhesion protein, vinculin, forms a complex intracellular linkage between the actin and the ECM [49,50]. CLSM images showing the immunostaining (Figure 4A,B) highlighted that the MG-63 cells attaching onto both pre-infected HTE-Ti and sterile AR-Ti surfaces were able to express vinculin and actin. Additionally, cells that were attached to the HTE-Ti surfaces were able to form tiny filopodia at the edges. These filopodia were absent on the cells attached to the AR-Ti surfaces. The filaments produced by the cells are known to serve as anchor points to increase the number of focal adhesion points [51]. The results of the BCA assay (shown in Figure 5A,B) suggest that the MG-63 cells that had attached onto the pre-infected HTE-Ti surfaces tended to express a greater amount of protein than the cells attaching to the AR-Ti surfaces. In addition to increased levels of protein expression, a qRT-PCR analysis also highlighted a greater upregulation of differentiation markers such as ALP, OCN, OPN, and Col 1 in cells that were attached to the HTE-Ti surfaces. This could explain the results obtained by the MTS and LDH assays, where the number of cells attaching to the HTE-Ti surface increased significantly on day 3 of incubation, compared to those attaching to the AR-Ti surface. At the same time, the results of the LDH assay, which provided a measure of cell damage, showed a significant decrease in the level of LDH on day 3 and day 5, suggesting that the cells had the capacity to repair themselves over the 5-day incubation period. These results could attributable to the upregulation of OPN in the cells grown on the HTE-Ti surfaces. OPN is known to have multiple physiological functions in osteosarcoma cells, including having been implicated in cellular attachment, proliferation, wound healing, and mineralization processes [52,53,54]. While OPN has few physiological functions apart from bone mineralization, ALP, OCN, and COL 1 are differentiation markers of osteoblast cells. The higher levels of these markers would indicate that the osteoblast cells have switched from being in proliferation phase to that of a differentiation stage. Although the exact functions of ALP and OCN markers are still being debated, they make up most of the ECM and affect the expression of other differentiation genes. A study by Tsao et al. showed that a knockdown of OCN could downregulate the expression of osteogenic markers, such as ALP and COL 1 [39,55,56,57,58]. Thus, bactericidal HTE-Ti surfaces have been shown to be able to promote attachment, proliferation and differentiation of MG-63 cells, whilst simultaneously killing pathogenic bacteria. It should be noted that in addition to hydrothermal etching, there are a number of other tools that could be used (on metallic and other types of implantable materials) to develop the desired nanoscale topography, providing scope for further optimization for the development of multicomponent implant devices [59] and other biomedical applications. For example, reactive plasma processing is an effective means for additive (e.g., surface growth) and subtractive (e.g., etching) modification of material surfaces [60,61], and fabrication of complex hierarchical topographies [62,63].

## 4. Conclusions

The results obtained in this study provide evidence that the nanostructured HTE-Ti surfaces exhibited not only bactericidal properties but also displayed biocompatibility towards MG-63 cell attachment, proliferation, and differentiation. There was no significant difference observed in the bioassay and gene expression results for the MG-63 cells grown on the HTE-Ti surfaces that had been pre-infected with *S. aureus* or *P. aeruginosa* cells. This provides further evidence that the HTE-Ti surfaces were able to specifically target the killing of pathogenic bacteria, whilst simultaneously allowing the MG-63 cells to grow and proliferate over the surface in a similar manner to that observed on the non-structured, smooth Ti surfaces. With an ageing population continuing to increase the demand for prosthetics and implants, prevention of implant-associated bacterial infections, especially from multidrug-resistant bacteria, is increasingly becoming a major international healthcare challenge. Given that nanostructured HTE-Ti has been shown to selectively eliminate bacterial contamination without the need for any antimicrobial agents, this bactericidal strategy represents a significant potential for application in the medical implant industry, as well as in many other areas where bacterial contamination is of significant concern, such as in the food processing and handling industry.

## Supplementary Materials

The following are available online at https://www.mdpi.com/1996-1944/12/10/1575/s1, Figure S1: Immunohistochemistry staining of proteins associated with growth and proliferation of MG-63 cells on AR-Ti. Vinculin, actin, and To-PRO3 were stained in green, red and blue, respectively. Cells were able to adhere to AR-Ti surface, as well as on pre-infected HTE-Ti surfaces. However, the cells on AR-Ti were not able to produce as many filopodia as the cells adhered to HTE-Ti surfaces.
