# Supplementary material for: The Fate of Osteoblast-Like MG-63 Cells on Pre-Infected Bactericidal Nanostructured Titanium Surfaces
Source: Materials (Basel). 2019 May 14;12(10):1575. doi: 10.3390/ma12101575 (PMC6567816; doi:10.3390/ma12101575)
Supplement: Supplementary file 1 [file materials-12-01575-s001.pdf]

materials

MDPI

Supplementary Information

The Fate of Osteoblast-Like MG-63 Cells on Pre-Infected Bactericidal Nanostructured Titanium Surfaces

Jason V. Wandiyanto [1], Vi Khanh Truong [2], Mohammad Al Kobaisi [1], Saulius Juodkazis [3], Helmut Thissen [4], Olha Bazaka [2], Kateryna Bazaka [5], Russell J. Crawford [2] and Elena P. Ivanova [2,*]

1 School of Science, Faculty of Science, Engineering and Technology, Swinburne University of Technology, Hawthorn, 3122 VIC, Australia; jwandiyanto@swin.eud.au (J.V.W.); makobaisi@swin.edu.au (M.A.K.)

2 School of Science, College of Science, Engineering and Health, RMIT University, Melbourne, 3000 VIC, Australia; vi.khanh.truong@rmit.edu.au (V.K.T.); olga.bazaka@rmit.edu.au (O.B.); russell.crawford@rmit.edu.au (R.J.C.)

3 Center for Micro-Photonics, Faculty of Science, Engineering and Technology, Swinburne University of Technology, Hawthorn, 3122 VIC, Australia; Sjoudkazis@swin.edu.au

4 CSIRO Manufacturing, Clayton, 3168 VIC, Australia; helmut.thiessen@csiro.com

5 Institute for Future Environments, Queensland University of Technology, GPO Box 2434, Brisbane, QLD 4001, Australia; katerina.bazaka@qut.edu.au

* Correspondence: elena.ivanova@rmit.edu.au

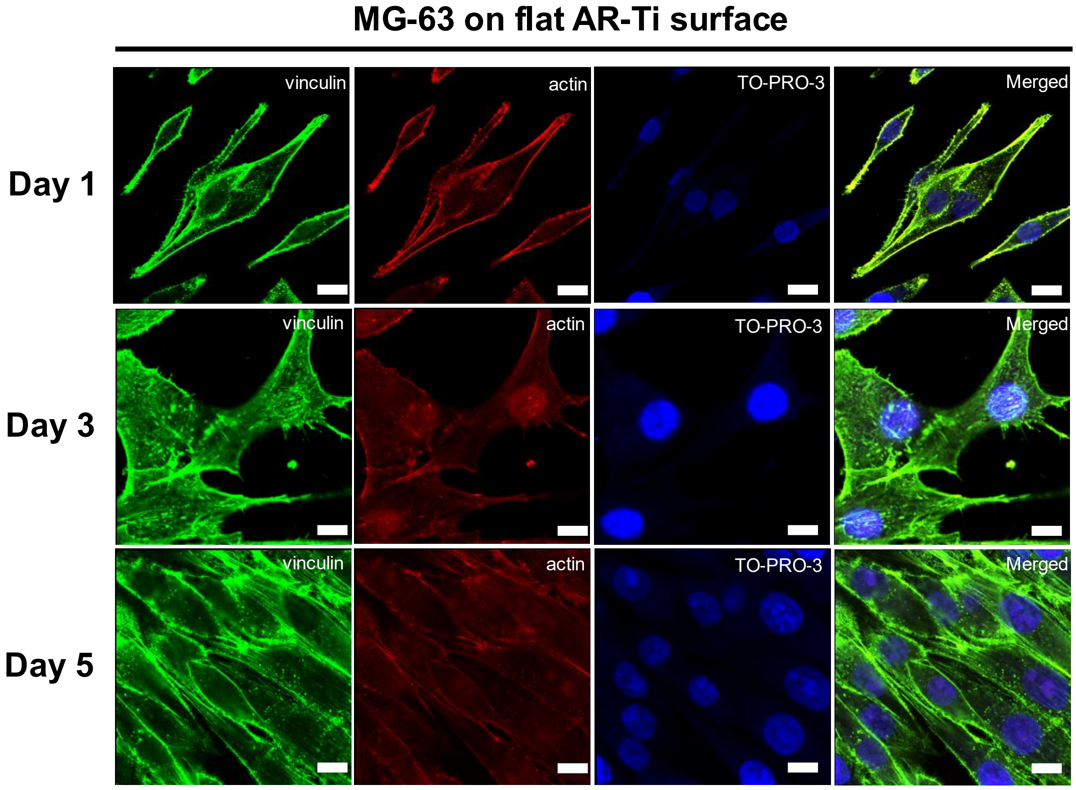

Figure S1. Immunohistochemistry staining of proteins associated with growth and proliferation of MG-63 cells on AR-Ti. Vinculin, actin, and To-PRO3 were stained in green, red and blue, respectively. Cells were able to adhere to AR-Ti surface, as well as on pre-infected HTE-Ti surfaces. However, the cells on AR-Ti were not able to produce as many filopodia as the cells adhered to HTE-Ti surfaces.
